# The Content of Mercury in Herbal Dietary Supplements

**DOI:** 10.1007/s12011-018-1240-2

**Published:** 2018-01-17

**Authors:** Barbara Brodziak-Dopierała, Agnieszka Fischer, Wioletta Szczelina, Jerzy Stojko

**Affiliations:** 1 Department of Toxicology and Bioanalysis School of Pharmacy with the Division of Laboratory Medicine, Medical University of Silesia, 4 Jagiellonska Str, 41-200 Sosnowiec, Poland; 2 Pharmacy, Blisko Ciebie”, 7A Więźniów Oświęcimia Str, 32-600 Oświęcim, Poland

**Keywords:** Herbal dietary supplements, Mercury

## Abstract

The dietary supplement market in Poland has been growing rapidly, and the number of registered products and their consumption increases steadily. Among the most popular and the easiest to get are herbal supplements, available in any supermarket. The aim of this paper was to investigate the mercury content in the herbal supplements. The dietary supplements that have been examined (24) are available on the Polish market and contain one or more herbal ingredients. Supplements were pulverized in porcelain mortar and identified by AMA 254 atomic absorption spectrometer. The range of variations for all tested supplements was within 0.02–4293.07 μg/kg. The arithmetic mean of the total result was 193.77 μg/kg. A higher mercury content then this mean was found in preparations—bamboo shoots and alga *Chlorella pyrenoidosa.* The studies have shown that mercury is present in every examined herbal supplement, and its content exceeds in two preparations (with bamboo and alga) the permissible limit of 0.10 mg/kg. There were statistically significant differences in the occurrence of mercury depending on the herbal ingredient in the supplement. The lowest content was found in the preparation with *Tanacetum parthenium* and the highest with bamboo shoots. The mercury content in the tested herbal supplements was statistically significant in the form of a supplement—a tablet and a capsule. Daily, weekly, monthly, and yearly consumption of mercury with examined supplements was calculated—the results did not exceed the PTWI—provisional tolerable weekly intake of mercury. To increase consumer safety, it is imperative to conduct further research on dietary supplements and implement a stricter quality control of the dietary supplements.

## Introduction

In the last few years, there has been an increase in the number of registered and consumed dietary supplements. According to research by the European Commission, in the years 1997–2005, the dietary supplement market in Poland has grown dynamically, and in this period, an increase of approximately 219% was noted [1]. A worrying problem is the misconception that a dietary supplement cures and has a similar effect to a medicinal product. There is a lack of reliable knowledge of the effects of supplements, possibilities of side effects, and interactions with other drugs and foods among patients. Increasingly popular are natural products made from plants, and the return to phytotherapy and traditional therapies is on the rise. However, substances contained in plant raw materials may show strong actions and interact with other medicinal products [2, 3].

A dietary supplement is significantly different from a medicinal product and should not be identified as one. Characteristic differences include registration: the registration of dietary supplements is a short and cheap process, and it is supervised by the Chief Sanitary Inspector. There is no research on the stability of supplements, its interactions with drugs and food, and the possible side effects. A dietary supplement can be put on the market by anyone, and according to the Supreme Chamber of Control, in 2016, there was about 30 applications per day. An important feature that distinguishes a supplement from a medicinal product is the labeling on the product packaging. A dietary supplement does not normally contain a leaflet required for a medicinal product [1, 4, 5].

Nearly 25% of US adults report concurrently taking prescription medication with a dietary supplement. Some supplements, such as St. John’s wort and goldenseal, are known to cause clinically important drug interactions and should be avoided by most patients receiving any pharmacologic therapy [6].

There are reports of mercury contamination of food products and dietary supplements. Mercury gets into the body over different ways depending on its form. According to the World Health Organization, mercury is considered as one of 10 chemicals that are the main health problem among people all over the world [7–9].

Mercury compounds show high toxicity to tissues and organs, both in humans and in animals. Mercury has an affinity for sulfhydryl groups of cell membranes. The toxic effect occurs by binding to membrane components [10]. Therefore, this element interferes with many enzymatic reactions, causes problems in migration and division of cells, and is responsible for cell damage, or even death [11–15]. The toxicity of mercury can also be explained by other mechanisms. This includes, among others, interactions with microtubules, enzyme inhibition, oxidative stress, and production of reactive oxygen species, as well as disturbance of protein and DNA synthesis [16].

Mercury induces a cytotoxic effect which reduces the amount of reduced glutathione and induces the production of free radicals. Mercury compounds are responsible for blocking enzymes involved in the repair of damaged DNA. They also lead to formation of chromosomal aberrations and changes in chromosome numbers. An important aspect is the penetration of mercury into the blood-brain barrier. Mercury can bind to lipids in the cell membrane, which leads to a change in membrane charge. There is a decrease in the efficiency of delivering active substances to the cell. This leads to its damage and death [16–19].

The aim of this manuscript is to assess the presence of mercury in herbal dietary supplements. The study was conducted to determine whether the actual amount of mercury contained in these supplements does not exceed acceptable standards. This analysis was the result of the growing interest in these preparations, increasing popularity of herbal substances, and the lack of quantitative and qualitative research on the composition of supplements before being placed on the market. The content of mercury in the tested supplements was also compared depending on the contained plant ingredient and the supplement form. The content of mercury delivered with supplements at different times of use was calculated as well.

A novelty in this research is the determination of mercury content in supplements, due to the increasing popularity and consumption of these products.

## Materials and Methods

The study material consisted of 24 diet supplements, which contained one or more vegetable raw materials. These were products available on the Polish pharmaceutical market and used as strengthening preparations that improve hair, skin, and nails; regulate glucose levels; and support slimming. The exact characteristics and composition of the products are given in Table 1. In the selected substances, there were also present vitamins and minerals. From each type of supplement, two pills or capsules were selected and hand-powdered in a porcelain mortar. Then, they were weighed and analyzed using the AMA 254 atomic absorption spectrometer. Approximately 20 mg of powdered dietary supplement was weighed for each analysis and labeled. The device was suitably cleaned in air and deionized water prior to each analysis, and blank tests were made.

**Table 1:** Characteristics of tested dietary supplements

No.	Herbal ingredient	Content in 1 tablet/capsule	Other ingredients	Form of supplement
1	Extract of barley shoots	440 mg	No data	Tablets
2	Horsetail, nettle	No data	Vitamin A, B1, B2, B6, B5, C, biotin	Tablets
3	Violet tricolor	150 mg	No data	Tablets
4	Extract of feverfew leaf	2,6 mg	Vitamin B5, B2	Tablets
5	Extraxt of artichoke leaves Rosemary leaf extract Extract of *Curcuma longa*	100 mg 125 mg 9 mg	No data	Tablets
6	Dong Quai ginseng extract Soy isoflavone extract Horsetail extract	50 mg 10 mg 50 mg	Vitamin A, D, E, C, B1, B2, B6, B12, PP	Capsules
7	Tricolor violet herb extract	no data	Zinc lactate	Tablets
8	Horsetail Nettle	75 mg 25 mg	Vitamin A, C, E, B1, B2, B6, B12, PP	Tablets
9	Horsetail extract Nettle extract	70 mg 54 mg	Vitamin C, B1, biotin, Zn	Tablets
10	Extract of horsetail stalks and leaves Nettle leaf extract Chicory inulin	50 mg 25 mg 120 mg	Vitamin A, C, B1, B2, B6, E, PP, biotin, Zn	Capsules
11	Horsetail extract Nettle extract	50 mg 27 mg	Vitamin C, B1, biotin, Zn	Tablets
12	Artichoke Black radish Mint Rosemary Chlorella	50 mg 30 mg 30 mg 30 mg 50 mg	Vitamin E, C, taurine, Zn	Tablets
13	Herb horsetail Nettle	220 mg 20 mg	Vitamin C, A, B1, B2, PP, L-cysteine, biotin, Si, Zn, barm	Tablets
14	Extract of millet Extract of wheat germ	50 mg 50 mg	Methionine, cystine, vitamin B6, B1, calcium pantothenate, Fe, Zn, Cu, barm	Capsules
15	Extract of horsetail herb	250 mg	Vitamin A, C, E, B1, B2, B6, B12, PP, Zn, I, Fe	Tablets
16	Horsetail extract Nettle extract	No data	Vitamin C, E, B1, B2, B6, B12, biotin	Capsules
17	Garcinia Cambogia (tamarynd Malabar HCA)	40 mg	Zn, Cr	Capsules
18	Extract of young barley Bitter orange fruit extract	350 mg 25 mg	Biotin, Cr	Tablets
19	Extract of white mulberry leaves *Gymnema sylvestre* leaf extract Extract of Ceylon cinnamon	250 mg 200 mg 101.25 mg	Zn, Cr	Tablets
20	Ginseng extract	60 mg	Vitamin A, D, K, C, E, B1, B2, B6, B12, Cu, Mg, Fe, Se	Tablets
21	Extract of horsetail Extract of bamboo shoots Extract of nettle herb	150 mg 50 mg 75 mg	Vitamin C, E, B1, B2, B6, B12, PP, folic acid, L-methionine, Fe, Zn, Cu, Se, I	Capsules
22	Green tea extract	50 mg	Vitamin B6, Zn, Cr	Capsules
23	Bamboo shoots Horsetail	85.72 mg 52.63 mg	Biotin, Zn, Cu, Mn, Se, Si, vitamin PP	Tablets
24	Alga *Chlorella pyrenoidosa* 100%	No data	No data	Tablets

Markings were made using the AMA 254 device. AMA 254 is an atomic absorption spectrometer that is designed to mark total mercury, regardless of the form in which mercury occurs. Mercury is easily released from its compounds (organic and inorganic) by being converted to an atomic form. This makes mercury marking simple and fast, and it is not necessary to use an atomic absorption spectrometer with a hydride generator attachment. Another advantage is that the pyrolytic mineralization process takes place inside the device, and it is not necessary to use the mineralizer for mercury marking.

Mercury measurement using this device consists of three stages:

- First, the solid or liquid sample is dried and then burned in a stream of oxygen.
- In the second stage, released mercury vapors pass through the catalytic column and are collected by the amalgamator (a small glass tube containing gold-coated ceramic material). After collecting all of the mercury from the released gases, the amalgamer is heated to about 900 °C, and mercury vapor is released to the detection system. In case of mercury-low matrices, it is possible to concentrate it more.
- At the third stage (detection), mercury vapor is segregated into two parts in a device called a cuvette. One part of the cuvette supplies a mercury carrier gas sample to an optical pathway conducive to low mercury concentration analysis, and the other part supplies gas to the optical pathway and is optimized for high mercury concentrations. This dual cuvette system allows the device to extend the dynamic range for analysis results at different mercury concentrations. The cuvette is set on the pathway of a typical atomic absorption spectrometer. The spectrometer has a mercury lamp that emits light at a wavelength of 253.7 nm and a silicon UV diode as a detector for mercury quantification [20].

This method uses the radiation absorption phenomenon by free mercury atoms in a basic state. It consists in the fact that free mercury atoms absorb the radiation emitted by the mercury lamp, the hollow cathode of which is made of mercury. As a result, the initial intensity of radiation emitted by the mercury lamp is reduced, and it is recorded by the spectrometer. The magnitude of this reduction is proportional to the number of mercury atoms in released pairs of this element.

The marking limit is 0.003 ng of mercury in the marked sample. Medical or technical oxygen that provides better combustion characteristics and guarantees repeatability of the measurement result is the carrier gas and the oxidizer.

The device is controlled by an external PC with advanced software (calibration curves, statistical analysis of results, process control with display of a current signal) running on Windows®.

The correctness of the applied method was determined using reference material INCT-MPH-2 Polish Herbs Mixture, in which the mercury-certified content was 0.018 ± 0.002 mg/kg. The content of mercury obtained from five repetitions was 0.019 ± 0.0004 mg/kg, and the recovery value was 105.6%.

### Statistical Analysis

The statistical analysis of the results obtained was made using Microsoft Excel and Statistica for Windows 12 pl.

The first stage of the statistical analysis of the results was to investigate the normality of mercury content distribution of the tested herbal supplements. For this purpose, the Shapiro-Wilk (W) test was used. The mercury content distribution deviated from a normal one (*p* > 0.05) and was right-side developed. Non-parametric tests were used for subsequent analyses. The values of arithmetic mean, standard deviation, change range, variation coefficient, and median were used to develop and describe the results.

Statistical variability between particular groups was based on the ANOVA rang Kruskal-Wallis test for multiple samples, and for equality between the two groups, the U Mann-Whitney test.

## Results

The lowest content of mercury was found in horsetail preparation No. 16 (0.07 μg/kg). A low content of mercury was also found in preparations: No. 4 with *Tanacetum parthenium* (0.55 μg/kg) and No. 6 multivitamin for women (0.59 μg/kg). The highest levels of mercury in the tested supplements were noted in preparations No. 23—with bamboo shoots (4.21 mg/kg) and No. 24 with algae (1.81 mg/kg).

The range of variations for all tested supplements was within the range of 0.02–4293.07 μg/kg. The arithmetic mean of the total result was 193.77 μg/kg. A higher mercury content then this mean was found in preparations 23 and 24 (Table 2).

**Table 2:** Statistical analysis of the content of mercury in herbal dietary supplements [μg/kg]

No.	AM	SD	Range
1	3.84	0.33	3.57–4.21
2	5.83	0.35	5.55–6.22
3	3.37	0.34	2.97–3.69
4	0.55	0.03	0.52–0.58
5	6.61	0.25	6.44–6.90
6	0.59	0.11	0.46–0.67
7	5.28	0.15	5.11–5.38
8	2.62	0.14	2.50–2.77
9	4.03	0.49	3.48–4.39
10	2.53	0.29	2.15–2.84
11	1.20	0.31	1.02–1.56
12	3.94	0.24	3.67–4.14
13	5.38	0.52	4.78–5.68
14	12.02	1.04	11.35–13.22
15	4.64	0.06	4.58–4.68
16	0.07	0.04	0.02–0.09
17	4.29	0.25	4.00–4.49
18	3.13	0.26	2.89–3.41
19	3.92	0.11	3.80–3.99
20	1.92	0.14	1.77–2.06
21	4.91	0.10	4.80–4.98
22	18.36	13.39	2.96–27.22
23	4212.04	114.59	4131.01–4293.07
24	1806.12	579.41	1193.41–2345.19
Total	*193.77*	*774.73*	*0.02–4293.07*

*AM* arithmetic mean, *SD* standard deviation

The distribution of mercury content in dietary supplements was not normal (*p* > 0.05) and was right-side developed. In the studied group, most of the results (40%) of mercury content ranged from 1 up to 10 μg/kg.

Among the tested supplements, there were such in which the plant ingredient was repeated. Therefore, the obtained results of the mercury content subjected to analysis depended on the plant material. Table 3 provides a statistical analysis of the mercury content depending on the type of the plant ingredient contained in the preparation.

**Table 3:** Statistical analysis of the content of mercury in herbal dietary supplements depending on the plant ingredient [μg/kg]

Herbal ingredient	AM	SD	Range
Barley shoots	3.48	0.47	2.89–4.21
Horsetail + nettle	3.59	2.00	0.02–6.22
Violet tricolor	4.19	1.05	2.97–5.38
Artichoke leaves	5.27	1.48	3.67–6.90
Ginseng	1.25	0.74	0.46–2.06
Chicory inulin	2.53	0.29	2.15–2.84
Feverfew leaf	0.55	0.03	0.52–0.58
Extract of millet	12.02	1.04	11.35–13.22
*Garcinia Cambogia*	4.29	0.25	4.00–4.49
White mulberry leaves	3.92	0.11	3.80–3.99
Green tea	18.36	13.39	2.96–27.22
Bamboo shoots + horsetail	1806.12	579.41	1193.41–2345.19
Algae	1687.76	2305.05	4.80–4293.07

*AM* arithmetic mean, *SD* standard deviation

The most common ingredient in the tested preparations was the combination of horsetail and nettles. This composition was found in eight supplements tested. The mean content of mercury in these preparations was 3.59 μg/kg. The lowest content was 0.55 μg/kg and it was in the preparation with *Tanacetum parthenium*. Of the 24 tested preparations, the highest content of mercury was found in the supplement that contained bamboo shoots + horsetail and 100% *Chlorella pyrenoidosa* algae. Statistically significant differences between the mercury content and the plant ingredient in the supplement were demonstrated using the Kruskal-Wallis ANOVA by ranks, and the significance level was *p* < 0.001 (Table 3).

The mercury content was analyzed according to the form of dietary supplement. Supplements were in the form of capsules and tablets. Tablets are characterized by a wide range of changes, whereas in capsules, a narrow range is observed. Using Mann-Whitney (U) test, significant differences (*p* < 0.05) between the occurrences of mercury depending on the form of the drug were shown.

An analysis of the hypothetical dosage of individual dietary supplements was made, and daily, weekly, monthly, annual mercury intake values were calculated taking into account the weight of the individual tablet/capsule (Table 4). The average daily intake of mercury with supplements was 0.34 μg, and the annual intake was 123.78 μg. The percentage of the provisional tolerable weekly intake (PTWI) for inorganic mercury was also calculated (Table 5.

**Table 4:** Statistical analysis of the content of mercury in herbal dietary supplements depending on their pharmaceutical forms [μg/kg]

Pharmaceutical forms	Number	AM	SD	Med	Range
Tablet	17	274.80	917.64	3.99	0.52–4293.07
Capsule	7	5.95	7.50	3.48	0.02–27.22

*AM* arithmetic mean, *SD* standard deviation, *Med* median

**Table 5:** The supply of mercury from dietary supplements for daily, weekly, monthly, and annual intake

No.	Daily max dosage (tablet/capsule)	Amount of Hg [μg/kg]	Weight of 1 tablet/capsule [mg]	Daily intake [μg]	Weekly intake [μg]	Monthly intake [μg]	Annual intake [μg]	%PTWI
1	2	3.84	620	0.00476	0.0333	0.143	1.74	0.83
2	2	5.83	700	0.00816	0.0571	0.245	2.98	1.43
3	2	3.37	540	0.00364	0.0255	0.109	1.33	0.64
4	1	0.55	620	0.00034	0.0024	0.010	0.12	0.06
5	4	6.61	620	0.01639	0.1147	0.492	5.98	2.87
6	1	0.59	1150	0.00068	0.0047	0.020	0.25	0.12
7	2	5.28	350	0.00370	0.0259	0.111	1.35	0.65
8	2	2.62	400	0.00210	0.0147	0.063	0.77	0.37
9	2	4.03	330	0.00266	0.0186	0.080	0.97	0.47
10	2	2.53	498	0.00252	0.0176	0.075	0.92	0.44
11	1	1.20	520	0.00063	0.0044	0.019	0.23	0.11
12	1	3.94	650	0.00256	0.0179	0.077	0.93	0.45
13	2	5.38	550	0.00592	0.0414	0.178	2.16	1.04
14	3	12.02	620	0.02235	0.1565	0.671	8.16	3.91
15	1	4.64	620	0.00288	0.0201	0.086	1.05	0.50
16	2	0.07	650	0.00009	0.0006	0.003	0.03	0.01
17	2	4.29	650	0.00558	0.0390	0.167	2.04	0.98
18	2	3.13	820	0.00513	0.0359	0.154	1.87	0.90
19	2	3.92	700	0.00549	0.0384	0.165	2.00	0.96
20	1	1.92	1200	0.00230	0.0161	0.069	0.84	0.40
21	1	4.91	600	0.00295	0.0206	0.088	1.08	0.52
22	2	18.36	220	0.00808	0.0565	0.242	2.95	1.41
23	1	4212.04	620	2.61	18.280	78.344	953.18	457.01
24	12	1806.12	250	5.42	37.93	162.55	1977.70	948.21
		254.81		0.34	2.37	10.17	30.52	59.34

## Discussion

Dietary supplements are foods intended to supplement a basic balanced diet. The increasing interest in supplements makes new products appear on the market all the time. According to the Supreme Audit Office, the dietary supplement market is developing very dynamically and it is expected that in the next years, it will grow by approximately 8% per year [1].

On the labels of dietary supplements, there is no information about possible side effects, contraindications, and interactions. This misleads the patient and suggests that a dietary supplement is a safe product. Irrational use of such products affects the human body negatively. This is due to the potential for drug overdose, drug interactions, or the effect on diagnostic tests [21–32].

Dietary supplement ingredients can be various substances. A large portion of supplements are vitamin and mineral products. Increasingly, people use products that contain herbal ingredients. The return to herbalism and traditional therapies is on the rise, and among people there is a widespread belief that an herbal product is a healthy one. However, this is a misconception because such products can be a threat to humans, interact with other drugs, and contain heavy metals such as Hg [28, 30, 33].

Mercury occurs naturally in the environment; it is a toxic element and can pose a threat to human health and life. The maximum level of mercury content in dietary supplements is set out in the Commission Regulation of 2 July, 2008, amending Regulation (EC) No. 1881/2006 that sets maximum levels for certain contaminants in foods. According to this regulation, the maximum level of mercury for dietary supplements is 0.1 mg/kg or 100 μg/kg [34]. Among 24 herbal supplements tested, 2 exceeded the permissible standards: No. 23–1806 μg/kg and No. 24 4212 μg/kg.

The number of published work on the content of mercury in herbal dietary supplements is not too big. According to Socha et al. [35] who studied mercury content in dietary supplements available on the Polish pharmaceutical market, none of the tested preparations exceeded the permitted standards. The range of changes was 0.10–47.99 μg/kg and was lower than the results. Korfali et al. [36] examined mercury content in supplements in Lebanon—this element was detected in all preparations but was at a minimum level and did not exceed acceptable standards. Italian researchers also dealt with the content of metals in dietary supplements [37]; in samples of herbal supplements, the mercury content was within the standard values [37]. In dietary supplements available in Croatia, mercury content was not exceeded [38]. However, there are reports of exceeded limits of mercury content, and this concerns herbal dietary supplements originated in Nigeria [39].

Comparing the results obtained with literature data, all are at a very similar level. According to Socha et al. [35], in food supplements containing horsetail and nettle, the average mercury content was 3.25 μg/kg, whereas in the results obtained in own work, 3.59 μg/kg. There are many reports in literature on the high content of Hg in green tea [40]. For mercury content in green tea, the results obtained were different. According to Socha et al. [35], the average mercury content in green tea was 3.25 μg/kg, and in our own work, it was 18.36 μg/kg. These differences may be due to the fact that there is a very large group of supplements with these herbal ingredients.

The studied supplements were in the form of capsules and tablets. Using Mann-Whitney (U) test, significant differences between the occurrences of mercury depending on the pharmaceutical form of the drug were shown. There is no information available in literature that could be of reference.

The last stage of the work was to take into account dosage of the supplement and the weight of a single tablet/capsule and to calculate daily, weekly, monthly, and annual intake of mercury with these preparations. The PTWI for mercury is 4 μg/kg body weight [41]. Two preparations exceeded the recommended standard value. However, it should be noted that on the packaging of the supplement, there is lack of reliable knowledge of the maximum duration of use. Overdosing of products and taking excessive doses is quite common because there is a belief that herbal products do not pose a life threat. In addition, polytherapy and combination of different drugs and supplements poses a risk not only in terms of interactions between preparations but also the maximum limit for mercury.

With regard to the results obtained, it can be stated that herbal supplements from Polish pharmacies mostly do not exceed the acceptable standards for mercury and do not pose a threat to consumers. Nevertheless, it should be kept in mind that there are still no specific regulations on dietary supplements and principles of their control and manufacture. More restrictive laws and the need for more research on dietary supplements are needed due to the health and safety of the entire population.

Consumers should pay more attention to the consumption of supplements. Authorities allowing supplements for human consumption should expand the number of tests before the introduction of the supplement on the market.

## Conclusions

The mercury content of the tested herbal supplements was in the range of 0.02–4212 μg/kg.

The stated mercury content in most herbal supplements tested did not exceed the acceptable standards.

There were statistically significant differences in the occurrence of mercury depending on the herbal ingredient in the supplement. The lowest content was found in the preparation with *Tanacetum parthenium* and the highest with bamboo shoots.

The mercury content in the tested herbal supplements was statistically significant in the form of a supplement—a tablet and a capsule.

To increase consumer safety, it is imperative to conduct further research on dietary supplements and implement a stricter quality control of the dietary supplements.
